# Immature Platelet Dynamics in Immune-Mediated Thrombocytopenic States

**DOI:** 10.3389/fmed.2020.597734

**Published:** 2020-12-18

**Authors:** Hollie M. Reeves, Robert W. Maitta

**Affiliations:** Department of Pathology, University Hospitals Cleveland Medical Center and Case Western Reserve University, Cleveland, OH, United States

**Keywords:** absolute immature platelet count, thrombotic thrombocytopenic purpura, immune thrombocytopenia, immature platelet fraction, immature platelets

## Abstract

A major challenge encountered by clinicians is differentiating presentations characterized by significant thrombocytopenia due to overlapping clinical symptoms and signs in the setting of ambiguous laboratory results. Immature platelets represent the youngest platelets that can be measured in peripheral blood by current hematology analyzers. These young platelets are larger, with higher RNA content recently released from the bone marrow. Thrombocytopenic presentations caused directly or indirectly by immune responses can lead to compensatory bone marrow responses seeking to normalize the platelet count; thus obtaining absolute immature platelet counts may be informative while triaging patients. Over the last decade, their use has expanded beyond being an early biomarker of bone marrow reconstitution post-hematopoietic stem cell transplantation to being used to establish bone marrow responses to infection and thrombocytopenias due to immune etiologies. Its accessibility as part of more detailed platelet indices obtained with routine laboratories makes it a promising option to understand the bone marrow's real-time response to disease states characterized by thrombocytopenia. This review will look at the immature platelet count as a biomarker, while presenting current attempts trying to understand how it could be used in thrombocytopenias occurring secondary to a given immune etiology.

## Introduction

Immunological processes that affect platelet production and/or platelet counts represent clinical challenges not only to diagnose but also to treat. Indeed, over the years a better understanding of some of these immune processes causing thrombocytopenia has led to more timely and targeted treatment approaches resulting in better outcomes. However, difficulties still remain when overlapping clinical pictures make a definitive diagnosis challenging. This can be exemplified by microangiopathic hemolytic anemia presentations where a given etiology may not be immediately apparent (1), and even in some instances when malignant states could be misdiagnosed as immune thrombocytopenia (ITP) (2). It is with this in mind that a growing body of literature describing potential alternative biomarkers lending support to a given diagnosis that results in timelier and etiology-specific interventions can be found. One of these markers, readily obtainable in modern automated hematology analyzers with fluorescence capability, is the immature platelet fraction (%-IPF) and specifically the absolute immature platelet count (A-IPC). Regardless of how the acronyms are presented in the literature, such as designation of either immature or reticulated platelets, it has become more evident that this is an important variable when discerning processes leading to a thrombocytopenic state which has long been overlooked in clinical practice (3–8).

Immature platelets have been shown to be much larger, with higher RNA content, and more biochemically active than their mature counterparts (9). They can be affected by chemotherapy treatments and irradiation, and when immune reactions target platelets they can be elevated well above reference ranges (9, 10). They can be accurately measured in blood samples even 24 h after they have been collected (11), likely due to their increased longevity compared to mature platelets (9). Additionally, consumptive thrombocytopenic processes and those characterized by platelet hypoproduction can be readily identified looking at immature platelets (12). These counts can potentially point out if the thrombocytopenia-inducing etiology is either centrally (at bone marrow) or peripherally driven (5, 13). Likewise, immature platelets appear not to be affected by gender (14) or age since their production is preserved even in older individuals with lower platelet counts (15).

Nevertheless, tests that have the potential to be used clinically need to undergo scrutiny that at times may come into a collision course with the constrains of the technology behind them. The number of reports describing the utility of immature platelet counts has been mostly positive favoring it as a gauge to zero-in on a narrower group of potential etiologies. Yet, technological differences exemplified by changes in dyes with improved specificity for platelet elements, changes in gating, size correction, testing platform used, and wavelengths influence the analytical limits of this test (16–18); while those factors that are pre-clinical in nature such as timing of measurement with respect to specimen collection, degree of platelet activation, anticoagulant used, and quality of the sample (degree of hemolysis) may begin to explain the finite discrepancies that appear in the literature (16, 19–22).

Measurement of immature platelet counts requires establishing better defined reference intervals (16, 17). Importantly, reports describing that A-IPC changes precede those of their mature counterparts at times by 2–3 days may be an important observation when treating patients with thrombocytopenia (23–25). This is also the case in patients recovering from chemotherapy or stem cell transplantation in which immature platelets are first to return indicating that engraftment has occurred (23, 26, 27). This is undoubtedly of benefit to predict patients engrafting or for those recovering from their immune-consumptive processes. This may also help even those patients whose bone marrow fails to produce platelets in sufficient numbers to compensate for an existing thrombocytopenic state (3). Finally, research into their biology is likely to become easier thanks to the development of newer assays that immunostain, sort, and isolate immature platelets from the peripheral circulation (28, 29). Thus, this is the focus of this review to present the most current information of potential uses of immature platelets counts in clinical practice.

## Immature Platelets in ITP

When the first data of changes in baseline %-IPF in the setting of ITP was reported, it raised the possibility that a real-time response by the bone marrow in this disease was possible to measure (30). Pathologically both higher platelet destruction that results in decreased platelet counts and potentially impaired thrombopoiesis influence risk of bleeding (31). Thus it should be of interest that reports appear to suggest that bone marrow attempts to compensate for platelet destruction by markedly increasing %-IPF to cope with the consumptive/destructive process (2, 8, 21, 30, 32–34); that these increases appear higher in those with chronic ITP (35); and that these dynamics may help risk stratify patients at risk of bleeding since they appear to have a higher preponderance of immature platelets (32, 36, 37). Notably, the magnitude of these compensatory increases in patients with ITP may be conveyed in a more consistent manner by looking at A-IPC at presentation (38).

**Figure 1:**
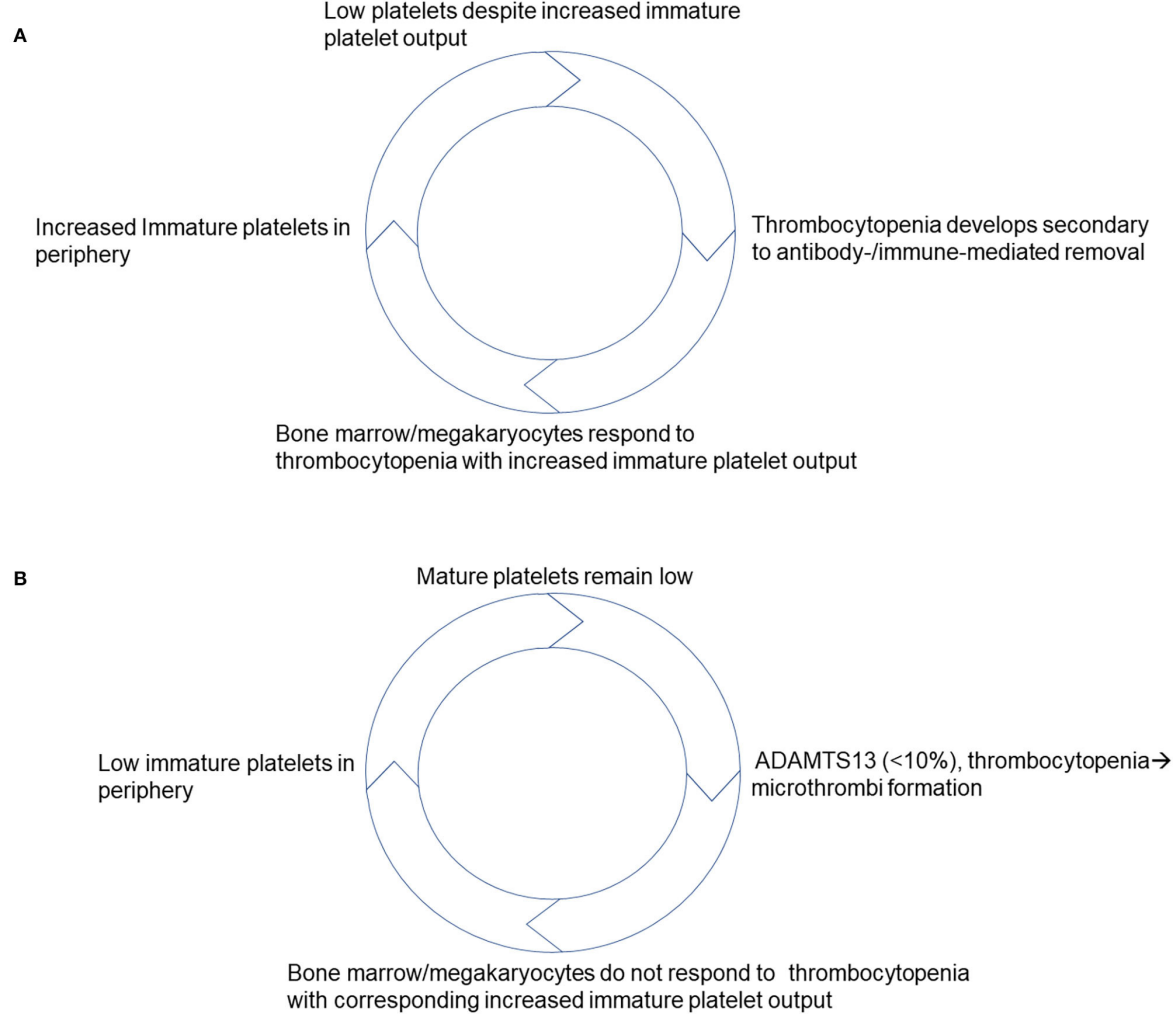
Schematic representation of proposed negative feedback models that take into account responses to thrombocytopenia based on immature platelet production. **(A)** Responses to thrombocytopenia in ITP. **(B)** Responses to thrombocytopenia in TTP.

However, few reports have found limited utility in using immature platelets to differentiate ITP where one hematology analyzer found some value to measuring it while the other had discrepant results (39). Regrettably, this study lacks A-IPC data analysis and determination if immature platelet counts were comparable with stratification according to platelet count (39). Despite this, recent data indicates that as ITP patients respond to treatment (40), specific changes in A-IPCs identify those with the disease (8). Based on these results, potentially helpful clinical scoring models that include immature platelet counts and favor ITP as a diagnosis have been proposed (2, 33), that take into consideration the high positive predictive value of these A-IPC changes (41). What adds to their potential usefulness is that in ITP, the higher immature platelet counts seen as patients recover from the disease (high turnover/destruction) precede by >3 days changes in mature platelet counts (25), similar to what has been described in other thrombocytopenic presentations. Thus, a model can be derived taking into account the published literature which favors a preserved and at times enhanced immature platelet/ mature platelet feedback (Figure 1A). In this model, as platelets get consumed or destroyed in the periphery, the bone marrow concomitantly increases its immature platelet output to compensate for platelet losses. Once platelet counts improve in response to disease remission and/or as a result of therapy, and platelet counts improve there is a corresponding decrease in immature platelet output as they return to baseline (10).

## Immature Platelets in Thrombotic Thrombocytopenic Purpura (TTP)

In the TTP literature, without a doubt the discovery that deficiency either innate or immune-mediated (acquired) of the metalloprotease ADAMTS13 is at the center of its pathology has changed the way in which patients are diagnosed and triaged (42, 43). This test, however, is sent-out to reference laboratories by most institutions often leading to initiation of therapeutic plasma exchange (TPE) prior to testing results becoming available. This is an important point to consider, since TPE is associated with higher adverse reactions when plasma is used as replacement fluid as required in TTP patients, making a timely accurate diagnosis paramount (44, 45). As a result, alternatives that increase the index of suspicion for TTP while not delaying prompt initiation of therapy could prove useful in such clinical settings. Early reports indicate that the immature platelet counts of TTP patients are much lower than those of ITP patients (30). Furthermore, recent data has shown that TTP patients (ADAMTS13 <10%) have A-IPCs at presentation that are markedly decreased compared to healthy controls that differentiates this group from other thrombocytopenic patients without the enzyme deficiency (24, 46, 47), and in some, such as refractory TTP cases, it may facilitate adjustments in therapy (48, 49). In TTP patients, improvement in A-IPC precedes that of mature platelet counts by about 2 days following initiation of TPE; and counts return to baseline once mature platelet counts stabilize at a normal level (24, 46, 50).

A-IPCs have been shown to predict response to TPE in patients with high ADAMTS13 inhibitors who may require longer treatment protocols to restore platelet counts (50). However, it remains to be determined if they would be helpful to assess responses of those TTP patients who are likely to require more TPE based on their ABO blood group (51). Patients with TTP as indicated by their immature platelet counts at presentation, appear to have a suppressed negative feedback i.e., rapidly relieved by TPE initiation (24, 46, 50). Based on this, we can propose a model in which an impaired immature platelet/ mature platelet negative feedback characterizes new onset TTP. This proposed model suggests that the bone marrow appears not to respond to the existing thrombocytopenia with a corresponding increase in immature platelets until TPE is initiated (Figure 1B). Nonetheless, post TPE initiation A-IPC increases, preceding the mature platelet count changes restoring the negative feedback. Once mature platelet counts reach a normal level, A-IPC begins to decline back to baseline indicating that patients are on their way to recovery. This apparent suppressed A-IPC response implies that the feedback mechanisms at play between mature and immature platelets are disrupted in new onset TTP. This should be the focus of future research looking at the mechanisms mediating this observation to establish how disease precipitates in TTP patients.

## Immature Platelets During Infectious Processes

Infectious processes may lead to decreases in mature platelets as the body fends off the infectious agent(s). Notably, even though thrombocytopenia can be seen in infections, A-IPCs are generally maintained so that at least platelet production attempts to keep up with the higher consumption (52); yet such A-IPC increases appear to correlate with higher mortality risk and disease severity in septic patients (53). These increases in immature platelets have been reported to occur earlier in patients prior to sepsis onset (54), which may be predictive of subsequent decreases in mature platelet counts once infection sets in Muronoi et al. (55). In this regard %-IPF has been reported as highly sensitive in identifying patients with sepsis regardless of extent of infection or severity (56, 57). This may relate to the significant immune hyperreactivity observed under states of severe infection leading to a disseminated platelet consumption requiring a higher immature platelet output. However, these increases may not be applicable to neonates where suppressed A-IPC characterizes those patients who did not survive disseminated infections (58). On the other hand, older children with dengue fever who recovered from the infection had increased immature platelet outputs up to 3 days prior to recovering their platelet count (59). Therefore, in an infectious presentation, the negative feedback between immature platelets and mature platelets appears generally preserved in older children and adults.

## Immature Platelet Counts in Inflammatory Settings

Inflammation-inducing disease processes that lead to impaired thrombopoiesis can be triaged looking at immature platelets as shown in patients with impaired liver function/cirrhosis (60). Similarly, states in which inflammation leads to platelet count changes can be ascertained looking at immature platelets. For example, higher counts of immature platelets in circulation may predict those patients at risk of subsequent inflammation post-cardiac surgery (61). Even 7 days after these surgical procedures a correlation between pro-inflammatory interleukin (IL)-6 and immature platelet counts has been reported, where the former is associated with the inflammation encountered by these patients (62). Potentially, these increases in immature platelets may be directly driven by IL-6 since this cytokine leads to thrombocytosis and platelet activation in intestinal inflammatory settings (63). However, the risk may be related to cardiovascular disease itself since human immunodeficiency virus (HIV) patients on antiretroviral therapy with cardiovascular disease have a higher number of immature platelets compared to HIV patients on therapy without cardiovascular disease (64).

Inflammation has also been shown associated with hypertension and this may lead the cardiovascular disease sequelae among other complications (65). For example, patients with malignant hypertension who present with thrombocytopenia have significantly higher immature platelet counts that are distinct from other microangiopathic hemolytic anemia processes including TTP (47, 66). Likely, sheer forces associated with hypertension lead to vascular damage and platelet consumption that result in a higher immature platelet output. Similarly, just as in other inflammatory processes it appears that the A-IPC allows for a better distinction between preeclampsia and those patients with hemolysis, elevated liver enzymes, and low platelet count syndrome (66). Along these lines, smoking causes vascular stenosis that lead to hypertensive complications and patients who are smokers have higher proportions of immature platelets (67). Paradoxically, some reports indicate that low grade inflammation may not provide enough of a stimulus to drive immature platelet production (68). Additional research is required to further characterize differences among these presentations.

## Immature Platelets and Drug-Induced Presentations

Immature platelets have been used to establish when a given drug has no effect over thrombopoiesis (69). Antibody-mediated reactions to complexes that include platelet factor four are mediated by use of heparin. Since platelets are affected by the presence of antibody to the PF4-heparin complexes, this can result in changes to immature platelet output. Mild increases in %-IPF have been reported in samples tested during heparin-induced thrombocytopenia (HIT) investigations (70). However, recently it was shown that patients who test positive (HIT^+^) for the presence of anti-PF4-heparin antibodies have A-IPC similar to the reference range unlike patients who test negative whose immature platelets are well-below this range (71). This implies that HIT^+^ patients may have immature platelet responses that attempt to maintain the platelet count though not necessarily leading to an increase in net immature platelet production. Future investigation should expand upon these observations.

## Concluding Remarks

Expansion over the last decade of the literature showing potential uses of immature platelet measurement in a variety of thrombocytopenic clinical settings (23, 38, 52, 71, 72), represents a promising development that has evidently resulted in heightened interest on their use.

Yet, it must be acknowledged that these promising reports, favor the establishment of well-controlled clinical trials that look at how immature platelet counts could affect disease management without compromising therapy timing (73). Likewise, as mentioned earlier, there are still remaining limitations since different analyzers will require establishment of reference ranges prior to potential application, and as technology advances with newer analyzers becoming available with higher specificity and sensitivity these ranges will undoubtedly require revision. Despite these apparent shortcomings, counts below, at, or above a reference range may prove clinically informative when discerning the etiology behind a thrombocytopenic presentation.

In summary, immature platelets are equivalent to reticulocyte counts in the setting of anemia and thus provide valuable information to the clinician when treating thrombocytopenic patients. These immature platelet counts provide the nearest to real-time information of bone marrow response to the etiology causing the thrombocytopenia, with the added benefit that once therapy is initiated it can guide a clinician to determine when therapy is causing a net shift in the production of these young platelets.

## Author Contributions

HR and RM contributed to drafting, editing, and finalizing manuscript to its current form. Both authors contributed to the article and approved the submitted version.

## Conflict of Interest

The authors declare that the research was conducted in the absence of any commercial or financial relationships that could be construed as a potential conflict of interest.
